# Early departures from the emergency department in a high-volume tertiary center: the first report from Croatia

**DOI:** 10.3325/cmj.2026.67.255

**Published:** 2026-08

**Authors:** Josip Krnić, Kristina Vidović, Petra Jerković, Benjamin Benzon

**Affiliations:** 1 Department of Emergency Medicine, University Hospital of Split, Split, Croatia; 2 Department of Anatomy, Histology, and Embryology, University of Split, School of Medicine, Split, Croatia; Krnić et al: Early emergency department departures in a high-volume tertiary center

## Abstract

**Aim:**

To determine the characteristics of early emergency department (ED) departures, including patients leaving without being seen and leaving during treatment, in a large tertiary center in Croatia.

**Methods:**

We retrospectively reviewed ED visits at University Hospital Center Split from July 12, 2023, to July 12, 2024. The study enrolled patients who had been triaged but left before physician evaluation or before completing diagnostic workup. Pediatric, gynecologic, psychiatric, and infectious disease ED visits were excluded because these specialties are managed separately. Data on demographics, Australasian Triage Scale category, referral type, specialty routing, seasonal/circadian patterns, and 48-hour return visits were extracted from the hospital information system.

**Results:**

Among 110 547 patients visiting the ED, 1420 (1.3%) departed early. Their median age was 40 years (interquartile range 25-59); 55.6% were male. Most were low-acuity patients (category 4: 60%; category 5: 29%). Early departures peaked in August-September and between 2 pm and 10 pm daily (maximum at 7 pm). Self-referred patients accounted for 65% of early departures, and 26% had been routed to orthopedics/trauma specialists. Only 5% left during treatment. Within 48 hours, 20% returned to the ED and 12% required other hospital evaluations.

**Conclusion:**

Early ED departure rate was low. The patients were mostly young, male, low-acuity, and self-referred with trauma-related conditions, presenting during tourist surges and evening hours. These findings underscore the need for dynamic staffing, enhanced triage communication, targeted patient education, and seasonal preparedness to sustain efficient ED performance.

Overcrowding in emergency departments (EDs) has major public health implications, affecting hospital throughput, staff capacity, and patient outcomes (1,2). Among the most immediate and measurable consequences of ED crowding is leaving without being seen (LWBS) (2,3). However, terminology across the literature is inconsistent. While many studies use LWBS as an umbrella term, the broader label “leaving before treatment is complete” (LBTC) includes both LWBS and leaving after treatment begins, as well as leaving against medical advice (LAMA) (4). Another approach distinguishes between LWBS and leaving during treatment (LDT), offering a more granular view of patient attrition (5).

All of these patients are directly affected by systemic congestion, and their early ED departures represent not only an individual health risk but also a broader failure of the health care system to meet urgent demand (1,6). Therefore, LWBS is more than a performance statistic; it represents operational inefficiency, patient dissatisfaction, and latent clinical risk. It also signals deeper systemic strain – breakdowns in communication, limited staffing, inefficient triage, and a lack of timely care (2,7).

LWBS rates worldwide range from 0.5% to over 20%, with higher figures in overstretched, high-volume EDs (3,8,9). Contributing factors include patient demographics, triage category, arrival time, staffing levels, and ED organization (3,8,10). Patients who fall into the LWBS category are typically young, male, and lower-acuity who leave after prolonged waits (3,5,9,11-14). Yet, this group is not clinically trivial: many return soon after with worsened conditions requiring hospitalization (2,6,15,16). Beyond clinical implications, LWBS burdens operations and finances: repeat visits strain staff, clog flow, and escalate costs (4,17).

LWBS is strongly influenced by perceived care quality. Prolonged waiting, inadequately communicated timelines, and visible crowding erode the trust of patients, especially those unfamiliar with triage protocols (7,18). LWBS trends are also shaped by circadian and weekly patterns. Incidents peak in late afternoon and evening, coinciding with high patient arrivals, reduced staffing flexibility, and longer waits (3,19-23). Among locals, low-acuity visits are often driven by limited primary care access outside clinic hours and work obligations. For non-residents, unfamiliarity with the system, language barriers, and discomfort further heighten frustration and likelihood of leaving (9). Overcrowding also affects the staff, contributing to burnout and impairing performance and care quality (24-26).

Particularly vulnerable to all these pressures are EDs in tourist-heavy regions such as Split. Each summer, the city experiences a substantial influx of non-resident patients who face linguistic, cultural, or systemic barriers that hinder communication and delay care. This seasonal patient surge increases the burden on triage and treatment workflows, correlating with elevated LWBS rates (9,27).

To the best of our knowledge, early ED departure rates have not been comprehensively studied in any Croatian ED. Therefore, this study assessed demographic profiles, temporal trends, specialty distribution, triage acuity, arrival patterns, and 48-hour readmissions of patients who departed early from a high-volume tertiary care center, spanning an entire year and capturing the seasonal impact of peak tourism. Our findings serve to inform evidence-based interventions aimed at optimizing ED operations, strengthening peak-season preparedness, improving patient satisfaction, and mitigating staff burnout.

## Patients and methods

### Study design and population

In this retrospective, single-center study, we reviewed the data related to 110 547 visits to the ED of the University Hospital Center (UHC) Split from July 12, 2023, to July 12, 2024. UHC Split is the largest tertiary institution in southern Croatia, affiliated with the University of Split School of Medicine, offering ~ 1400 inpatient beds and comprehensive specialty services. The ED, a high-acuity, high-volume entry point, receives patients from a wide regional catchment area, including a seasonal tourist influx. It is equipped with 30 acute care beds for stabilization and management of critically ill and injured patients, providing multidisciplinary care and serving as a pivotal node in the regional emergency network.

### Organization and workflow

Upon arrival, patients undergo standardized triage by a trained nurse. A structured questionnaire is used inquiring about symptoms, vital signs, medical history, and condition. Categorization follows the five-level Australasian Triage Scale (ATS): 1 – immediate resuscitation; 2 – life-threatening, assessment and treatment within 10-minute; 3 – urgent, assessment and treatment within 30-minute; 4 – semi-urgent, assessment and treatment within 60-minute; and 5 – non-urgent, assessment and treatment up to 120-minute (28). Following triage, patients are routed to subspecialty ED units based on symptoms or suspected pathology. When uncertain about the appropriate further department, the triage nurse immediately consults the on-duty ED specialist, who guides patient placement based on clinical judgment.

In this study, early ED departures were operationally defined as a composite outcome including both LWBS and LDT. All patients later classified as early ED departures had an ATS category assigned at presentation. The ED at UHC Split operates two main entry points: surgical medicine (trauma, orthopedics, neurosurgery, plastic surgery, general surgery, ophthalmology, and otorhinolaryngology) and internal medicine (cardiology, pulmonology, neurology, gastroenterology, endocrinology, nephrology, hematology, and rheumatology). Despite this dual access, pathways remain unified through standardized triage and coordinated oversight.

### Data collection

Patient data were retrospectively extracted from the hospital information system (HIS) of the UHC Split. The study enrolled all patients who had undergone triage but left before being evaluated by a physician or during treatment. Patients presenting directly to gynecologic, psychiatric, infectious disease, or pediatric EDs were excluded, as these constitute separate specialty-specific EDs. Children under 18 were included only if assessed within the core ED’s surgical division – pediatric surgery, an integrated subspecialty of the ED.

### Study variables and measurement instruments

The following variables were collected: age; sex; ATS triage category; seasonal distribution (monthly stratification over one year); ED arrival time (hour of the day); referral physician (self-referred, primary care physician, ED specialist, or other health care provider); ED specialty-based emergency unit (eg, internal medicine, surgery, pediatrics, neurology); whether the patient initiated the diagnostic workup before leaving the ED; whether the patient returned within 48 hours after leaving the ED.

The study was approved by the Ethics Committee of the UHC Split and conducted in accordance with the Declaration of Helsinki and national data protection legislation. Due to the retrospective nature of the analysis and full anonymization of patient data, individual informed consent was not required.

### Statistical analysis

Continuous variables are expressed as means ± standard deviations or medians and interquartile ranges, and categorical variables are expressed as counts and percentages. Differences in continuous data were assessed with a Mann-Whitney test, and differences in categorical data with a Pearson χ^2^ test or Cochran-Armitage χ^2^ test for trend. *P* values less than 0.05 were considered significant. Data were visualized and analyzed with Microsoft Excel and Python 3.12.3 (Python Software Foundation, Wilmington, DE, USA) using pandas 3.0.2, NumPy 2.4.4, and SciPy 1.17.1, with openpyxl 3.1.5 for spreadsheet handling. Analysis code was generated with Claude Opus 4.7 (Anthropic, San Francisco, CA, USA), and its output was reviewed and verified by one of the authors (B.B.).

## RESULTS

In the study period, 110 547 patients visited the ED of the UHC Split. Among these, 1420 were classified as early ED departures, which represented an annual incidence rate of 1.3%. The median age of these patients was 40 years (IQR 25-59), with an overall age range spanning 1 to 98 years. Male patients accounted for 55.6% of all cases.

**Figure 1:**
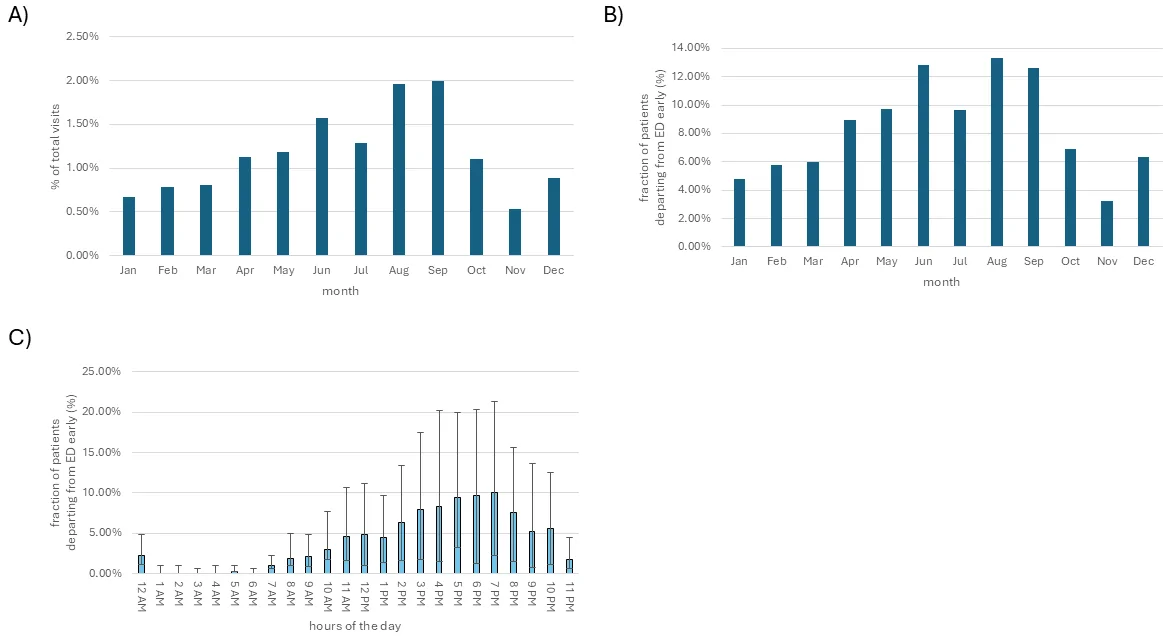
Temporal distribution of early emergency department (ED) departures at University Hospital Center Split, July 2023 – July 2024. (**A**) The monthly rate of early ED departures expressed as the percentage of all ED visits per month, showing a clear seasonal peak in August and September ( ≈ 2.0%) and a nadir in November (0.5%). (**B**) Distribution of all early ED departures across calendar months as a proportion of the full cohort, demonstrating that approximately one-quarter of cases occurred during the August-September summer tourist peak. (**C**) Hourly distribution of early ED departures across the 24-hour cycle, with bars representing the median (IQR) proportion of departures occurring in each hour and error bars indicating variability across study days. Early ED departures concentrated between 2 pm and 10 pm, peaking at 7 pm

The early departure rates peaked in August and September, collectively accounting for 2% of all ED visits that month and comprising 25% of annual early ED departures (Figures 1A and 1B). The highest rate was observed between 2 pm and 10 pm, with a peak at 7 pm (Figure 1C).

**Figure 2:**
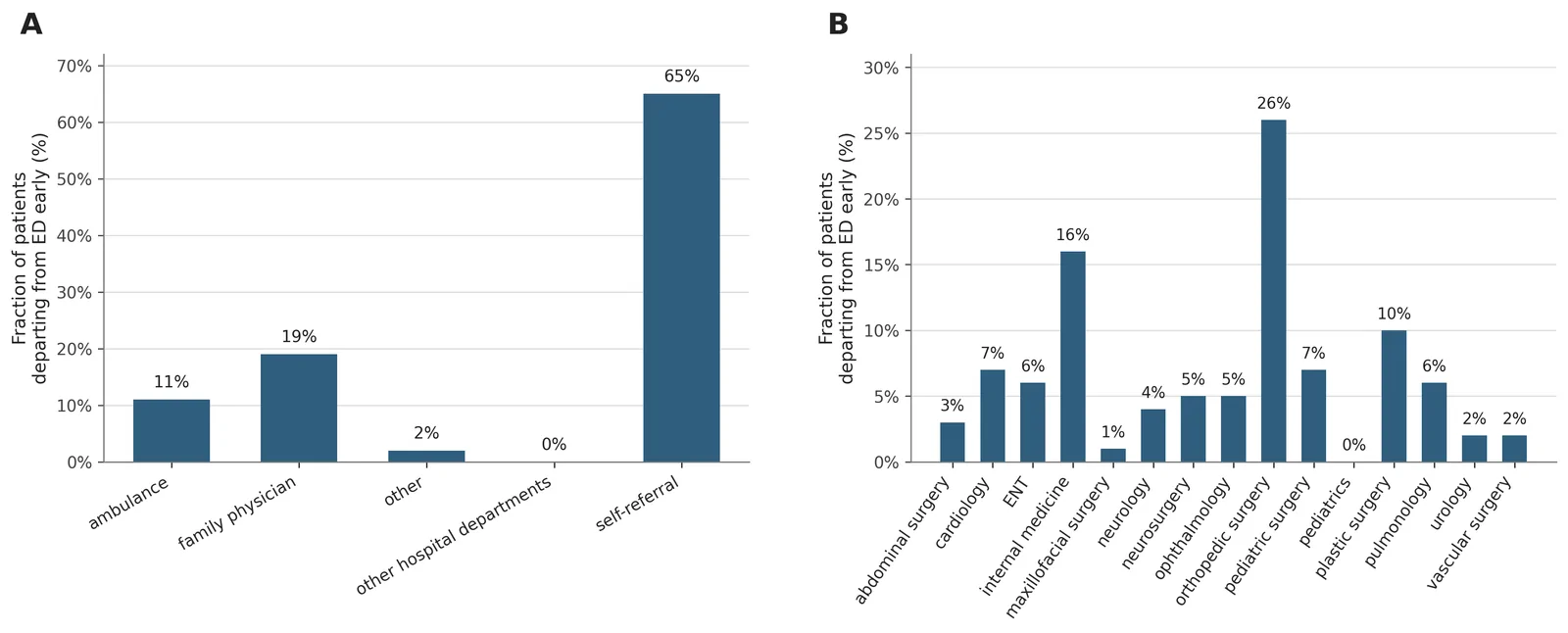
Distribution of patients with early emergency department (ED) departure by source of referral (3% are missing data) (**A**) and assigned specialty (**B**).

The majority of early departing patients (65%) were self-referred, 19% were referred by family physicians, and 11% by ambulance services. The remaining patients (2%) were referred via alternative pathways (Figure 2A). The highest proportion of early departing patients were routed to orthopedic/trauma surgeons (26%), followed by general internal medicine consultants (16%) and plastic surgeons (10%) (Figure 2B).

The majority of early departing patients were classified as low-acuity cases, with 60% assigned to triage category 4, and 29% to category 5. Moderate-acuity cases (category 3) accounted for 10%, while high-acuity cases (categories 1 and 2) comprised less than 1% of all early ED departures.

Among early departing patients, only 5% started diagnostic workup or physician assessment before leaving (LDT), while 95% left before any medical evaluation (LWBS). Of 1420 early departing patients, 288 (20.3%) returned to the ED and 170 (12.0%) were subsequently evaluated at another hospital department, including 76 patients (5.4%) who experienced both. Overall, 382 patients (26.9%) had a documented follow-up health care contact. When both returning and non-returning patients were stratified by ATS, we noticed an interesting linear trend of a monotonic increase from category 3 (20.8%) to category 5 (30.9%) (Cochran-Armitage trend test, z = 2.71, *P* = 0.007, Table 1). Furthermore, when returning patients were analyzed by specialty, a significant trend was noticed (χ^2^ = 35.49, df = 14, *P* = 0.0012, Table 2). The return rate was highest for vascular surgery (53%), followed by urology (41.9%) and pulmonology (39.5%); on the other hand, cardiology (19%) and neurosurgery (13.6%) had the lowest return rates. The returning patients were significantly older (median 45, IQR 29-67 years) than non-returners (38, 24-57 years) (Mann-Whitney U = 216 280, *P* < 0.001). Moreover, return rates increased monotonically with age category, from 22.1% in patients aged 18-29 to 37.0% in those ≥75 (χ^2^ = 16.95, df = 5, *P* = 0.005, Table 3).

**Table 1:** No. (%) of patients who returned and those who did not return after early emergency department (ED) departure according to triage category*

	Non-returners^†^	Returners^†^	Total	Return rate^‡^
**Triage category** Australasian Triage Scale)	n = 1036	n = 382	N = 1418	(%)
1 – Resuscitation	1 (0.1)^§^	0 (0.0)	1	0.0
2 – Emergent	3 (0.3)	0 (0.0)	3	0.0
3 – Urgent	114 (11.0)	30 (7.9)	144	20.8
4 – Semi-urgent	623 (60.2)	221 (57.9)	844	26.2
5 – Non-urgent	293 (28.3)	131 (34.3)	424	30.9

*Cochran-Armitage χ^2^ test for trend (all 5 categories): *z* = 2.711, *P* = 0.007.

†Returners are defined as patients who returned to the ED within the follow-up window or who were subsequently evaluated at another hospital department for the same complaint. Non-returners are patients with a documented absence of both follow-up types. Two patients with ambiguous follow-up data and two patients with missing triage information were excluded.

‡Return rate is calculated row-wise as returners/total ×100.

§Numbers in brackets are the percentages of each ATS category within returners and non-returners groups.

**Table 2:** No. (%) of patients who returned and those who did not return after early emergency department (ED) departure according to referral specialty*

Specialty^§^	Total	Non-returners^†^	Returners^†^	Return rate^‡^
	n	n	n	% (95% confidence interval)
**Surgical specialties**				
vascular surgery	26	12	14	53.8 (35.5-71.2)
urology	31	18	13	41.9 (26.4-59.2)
abdominal surgery	36	23	13	36.1 (22.5-52.4)
plastic surgery	144	99	45	31.3 (24.2-39.2)
ophthalmology	71	49	22	31.0 (21.4-42.5)
pediatric surgery	100	73	27	27.0 (19.3-36.4)
orthopedic/trauma	372	278	94	25.3 (21.1-29.9)
otorhinolaryngology	86	65	21	24.4 (16.6-34.5)
maxillofacial surgery	13	10	3	23.1 (8.2-50.3)
neurosurgery	66	57	9	13.6 (7.3-23.9)
Surgical subtotal	945	684	261	27.6 (24.8-30.6)
Medical specialties				
pulmonology	81	49	32	39.5 (29.6-50.4)
neurology	51	37	14	27.5 (17.1-40.9)
internal medicine	227	174	53	23.3 (18.3-29.3)
cardiology	105	85	20	19.0 (12.7-27.6)
pediatrics	2	2	0	0.0 (0.0-65.8)
Medical subtotal	466	347	119	25.5 (21.7-29.7)
Total	1411	1031	380	26.9 (24.6-29.3)

*Pearson χ^2^ test for heterogeneity across all 14 specialties: χ^2^ = 35.49, *df* = 14, *P* = 0.0012. Monte Carlo simulation (10 000 iterations, conditioned on row and column margins): *P* = 0.0018. Surgical vs medical division (2 × 2 comparison): χ^2^ = 0.59, df = 1, *P* = 0.444; odds ratio 1.11 (95% confidence interval 0.87-1.43).

†Returners are defined as patients who returned to the ED or were subsequently evaluated at another hospital department for the same complaint within the follow-up window. Non-returners are patients with a documented absence of both follow-up types. Two patients with ambiguous follow-up data and seven patients with missing specialty data were excluded from the analysis.

‡Return rate is calculated row-wise as returners/total ×100, with 95% confidence intervals computed using the Wilson score method.

§Specialty allocation between surgical and medical divisions follows the institutional emergency department structure.

**Table 3:** No. (%) of patients who returned and those who did not return after early emergency department (ED) departure according to age*^†^

Age (years)	Total	Non-returners^†^	Returners^†^	Return rate^‡^
	n	n	n	% (95% confidence interval)
<18	154	119	35	22.7 (16.8-30.0)
18-29	299	233	66	22.1 (17.7-27.1)
30-44	314	229	85	27.1 (22.5-32.2)
45-59	266	192	74	27.8 (22.8-33.5)
60-74	209	137	72	34.4 (28.3-41.1)
≥75	127	80	47	37.0 (29.1-45.7)

*Cochran-Armitage χ^2^ test for trend across age categories: χ^2 ^trend = 15.38, df = 1, *P* < 0.001 (z = 3.92).

^†^Returners are defined as patients who returned to the ED or were subsequently evaluated at another hospital department for the same complaint within the follow-up window. Non-returners are patients with a documented absence of both follow-up types. Two patients with ambiguous follow-up data and 49 patients with missing or invalid age values were excluded from the analysis.

‡Return rate is calculated row-wise as returners/total ×100, with 95% confidence intervals computed using the Wilson score method.

## DISCUSSION

The early departure rate observed in our study (1.3%) reflects effective operational capacity in our ED and is consistent with globally observed LWBS rates (0.49% to 20%) (3,8,9). While this rate positions our ED within the lower-risk bracket for both LWBS and LDT, it represents 1420 patients annually, each at risk of delayed care, repeat ED visits, and adverse outcomes (2,4,6,15-17).

The demographic profile of LWBS patients highlights trends consistent with the existing literature: younger, working-age patients, typically healthier and independent, but less tolerant of waiting and greater time constraints (3,5,9-14,22,29).

Men comprised 55.6% of LWBS cases. While some previous studies have identified male sex as a predictor of early departure (5,10,19,30,31), others reported a balanced sex distribution or female predominance (1-3,9,22,23). Male predominance can be explained by several factors: behavioral tendencies toward risk minimization, emotional response to waiting, sociocultural expectations, “stoicism” as a desirable trait in men, greater likelihood of LAMA, greater substance use, reduced help-seeking behavior, and higher impulsivity under stress (10,30,31).

A substantial proportion of early departing patients had been routed to orthopedic and trauma surgery. This finding likely reflects musculoskeletal complaints – minor injuries or pain syndromes, which are rarely perceived as life-threatening and are deprioritized in triage (29,32,33). Moreover, the predominance of trauma-related presentations also reflects environmental and occupational factors, particularly among younger men engaged in physical labor, sports, or high-risk activities. These findings mirror prior literature identifying trauma and musculoskeletal complaints, together with internal medicine complaints, as leading contributors to LWBS (3,5,10,22,29).

Our study revealed a distinct circadian pattern, with early ED departures peaking between 2 pm and 10 pm, most prominently around 7 pm. This coincides with cumulative daily patient arrivals reaching critical capacity. Other studies also reported late afternoons and early evenings to be high-risk periods for patient disengagement (3,19-23). This temporal clustering indicates structural misalignment between patient demand and staffing. Many EDs continue to operate on fixed staffing schedules that fail to accommodate predictable surges in daily arrivals, causing workflow bottlenecks and reduced efficiency (34-36).

Most early departing patients were triaged as low-acuity (categories 4 and 5), a finding consistent with previously reported trends (3,5,9,11-14). These patients are often hemodynamically stable and leave the ED, driven more by perceived waiting times and communication (7,18). Although low-acuity patients may not need immediate care, they might require guidance as their conditions could worsen or be underestimated. Importantly, the presence of higher-acuity patients (triage categories 2 and 3) among LWBS cases in our study, although minimal, underscores the importance of maintaining efficient triage processes. In previous studies, 4%-10% of LWBS cases fell into the intermediate-to-high acuity categories, which highlights the importance of minimizing delays and maintaining triage vigilance (4,10).

Our findings reflect the dual burden of Split being both a regional medical hub and a major tourist destination. Early ED departures were highest in August and September, likely due to the peak tourist season, consistent with patterns in other tourist-heavy EDs (5,9,23,27,29,32,37,38).

Notably, only 5% of early ED departures were LDT, which indicates that most patients left very early in the care pathway. This underscores the importance of the initial patient experience in shaping the decision to leave, especially waiting time before the first provider contact (5). Approximately one quarter (26.9%) of early ED departures had a documented subsequent health care contact, with 20.3% returning to the ED and an additional 12.0% being evaluated in other hospital departments. These findings are consistent with previously reported LWBS return rates of 15%-30% (4,5,10,22,39). On average, older patients returned more frequently.

About 65% of early ED departures were self-referred, which aligns with prior studies showing that patients bypassing primary care often have a limited understanding of ED workflows (5,9). These patients often seek reassurance or rapid evaluation but become frustrated when confronted by long waits, regardless of urgency (34-36).

This study suffers from limitations inherent to a retrospective study. Conducted at a single high-volume tertiary center in an urban setting with universal health care, findings may not be generalizable to rural systems, fragmented access models, or privatized frameworks. Moreover, we relied exclusively on the HIS, which, although robust, does not capture patient-reported reasons for departure, dissatisfaction with waiting times, language barriers, or perceived urgency. Additionally, due to inability to track individuals leaving before triage or registration, the study likely underestimated the true scale of disengagement. Operational definitions of LWBS also vary. We referenced internationally accepted standards, but some authors classify only patients leaving after triage but before physician contact, while others include mid-treatment departures (4,5). Relevant early ED departures might have been missed due to the exclusion of patients presenting to gynecology, infectious disease, psychiatric, and pediatric EDs. Outcomes were restricted to 48-hour ED return visits, leaving longer-term trajectories – hospitalizations, complications, or mortality – unexplored. This restricted the evaluation of downstream clinical consequences and safety, highlighting the need for future prospective studies with longer follow-up.

In conclusion, the low annual early ED departure rate, including LWBS and LDT, in our study could be attributed to several local operational protocols. We emphasized dynamic shift scheduling, reinforcing staff between 10 am and 10 pm, when crowding was most pronounced. An additional physician was assigned on Mondays and Fridays, traditionally the busiest days due to primary care referrals. Staffing was further increased during the summer tourist surge. At peak congestion, patients were quickly initiated into diagnostic workup (sent to blood sampling or basic tests), which ensured a rapid beginning of evaluation and maintaining engagement before full assessment. Clear communication between triage nurses and physicians was continuously reinforced, supported by the use of HIS tools such as real-time status displays and automated alerts to enhance transparency for both staff and patients.

Building on these strategies, several avenues for further improvement are evident. A formalized fast-track system for low-acuity complaints could relieve bottlenecks and preserve capacity for higher-acuity cases. Systematic patient feedback could guide service improvements and highlight the value of targeted communication and provider-in-triage models, ensuring early clinical contact (34-36). Closer coordination with primary care, especially on high-volume days, may reduce inappropriate referrals (5,9). Finally, proactive seasonal planning, including the preparation of multilingual materials and tailored patient guidance, could strengthen preparedness for the tourist influx (34-36). Together, as emergency care becomes more complex and patient populations more heterogeneous, these initiatives may support maintaining low early departure rates and further optimize ED performance.
